# Comparative analysis of *TP53* gene in *Tupaia belangeri* subspecies (*Tupaia belangeri yaoshanensis* vs. *Tupaia belangeri chinensis*) and identification of mutations in spontaneous tumor cases

**DOI:** 10.1002/ame2.70260

**Published:** 2026-08-17

**Authors:** Yingying Cao, Haibo Tang, Liang Liang, Jing Leng, Weijian Huang

**Affiliations:** ^1^ Guangxi University Nanning China; ^2^ Guangxi University of Chinese Medicine Nanning China; ^3^ Key Laboratory for Complementary and Alternative Medicine Experimental Animal Models of Guangxi Nanning China; ^4^ Guangxi Key Laboratory of Translational Medicine for Treating High‐Incidence Infectious Diseases with Integrative Medicine Nanning China; ^5^ Laboratory of Animal Infectious Diseases and Molecular Immunology, College of Animal Science and Technology Guangxi University Nanning China; ^6^ Guangxi Zhuang Autonomous Region Engineering Research Center of Veterinary Biologics Nanning China; ^7^ Guangxi Key Laboratory of Animal Breeding, Disease Control and Prevention Nanning China

**Keywords:** cancer model, sequence variants, spontaneous tumors, subspecies variation, *TP53*, tree shrew

## Abstract

The tree shrew (*Tupaia belangeri*) is an emerging animal model for human diseases, yet the status of the critical tumor suppressor *TP53* in its subspecies and spontaneous tumors is unknown. We sequenced the entire *TP53* coding region from two subspecies, *T. b. yaoshanensis* and *T. b. chinensis*, and analyzed two spontaneous sarcomas from *T. b. yaoshanensis* using histopathology, immunohistochemistry, and cDNA sequencing with clonal validation. *TP53* showed 99.5% nucleotide identity between subspecies, with a single fixed non‐synonymous substitution (D42G) in *T. b. yaoshanensis* predicted to be functionally neutral. All residues corresponding to human cancer hotspots were strictly conserved, and structural modeling confirmed high similarity to human p53 (RMSD = 0.6 Å). Critically, we identified *TP53* sequence variants in both spontaneous tumors: a missense variant (Ser175Gly) in the DNA‐binding domain of a liposarcoma and another (Met344Thr) in the tetramerization domain of a myxofibrosarcoma. Computational prediction suggested both missense variants are tolerated (SIFT scores 0.61 and 0.77), and their functional significance remains to be determined. This study provides preliminary evidence of *TP53* variants in spontaneous tree shrew tumors and reveals natural subspecies variation. The high conservation of p53 reinforces the tree shrew's relevance as a model for *TP53*‐related cancer research, although further functional studies are needed.

## Graphical abstract

This study provides the first evidence of natural *TP53* variation between tree shrew subspecies and identifies somatic *TP53* mutations in spontaneous tree shrew sarcomas. The high structural and functional conservation of tree shrew p53 with humans supports its utility as a relevant model for *TP53*‐related cancer research.

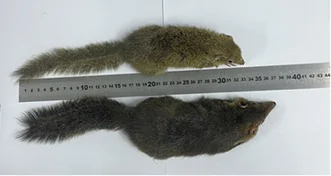

## 1 INTRODUCTION

The tree shrew (*Tupaia belangeri*), a small mammal occupying a unique phylogenetic position between primates and rodents, has emerged as a promising model for human biomedical research. Its high genetic affinity to humans, small size, rapid reproductive cycle, and susceptibility to human pathogens and metabolic diseases underscore its utility.^1^, ^2^ Notably, tree shrews exhibit advanced neuroanatomical features (e.g., lissencephalic brain) and cognitive behaviors comparable to primates, making them uniquely suited for studies of viral infections (e.g., hepatitis B/C, herpesviruses), depression, myopia, and metabolic diseases.^3^, ^4^, ^5^, ^6^, ^7^ Recently, the tree shrew's potential in oncology has been highlighted, with studies demonstrating its susceptibility to carcinogen‐induced hepatocarcinogenesis and the conserved function of key tumor suppressor pathways, suggesting its untapped value as a cancer model.^8^, ^9^

Encoding the p53 protein—the celebrated “guardian of the genome”, the tumor suppressor gene *TP53* plays a fundamental role in suppressing tumor development. P53 orchestrates cellular responses to DNA damage, oncogenic stress, and hypoxia by activating DNA repair, cell cycle arrest, senescence, or apoptosis.^10^ Mutations in *TP53*, predominantly within its DNA‐binding domain (DBD, exons 5–8), occur in >50% of human cancers and are associated with poor prognosis and therapeutic resistance.^8^ The functional conservation of p53 across mammals underscores its value in comparative oncology. In the tree shrew, initial characterization of the *TP53* gene revealed high sequence homology with humans, particularly within the DBD, suggesting conserved tumor‐suppressive functions.^11^, ^12^ Nevertheless, whether *TP53* mutations drive spontaneous carcinogenesis in tree shrews remains unexplored—a critical gap, given their increasing use in cancer modeling.^9^, ^11^ The study of spontaneous tumors, which arise without experimental induction, is particularly valuable for understanding natural cancer biology and validating the relevance of an animal model to human disease.^13^, ^14^, ^15^, ^16^

*Tupaia belangeri* comprises multiple geographically segregated subspecies exhibiting morphological, physiological, and genetic divergence. Two subspecies with significant research utility are the *Tupaia belangeri chinensis* (*T. b. chinensis*) from Yunnan/Sichuan and the *Tupaia belangeri yaoshanensis* (*T. b. yaoshanensis*) from Guangxi.^17^ Phylogenetic analyses based on complete mitochondrial genomes place these two subspecies in distinct terminal branches, confirming their significant genetic differentiation.^18^, ^19^ However, whether inter‐subspecies variation in *TP53* exists, and how it might influence cancer susceptibility or model validity, remains entirely unexplored. Compounding this knowledge gap, spontaneous tumors in tree shrews are rarely documented, with only a limited number of cases reported in the literature, including hepatocellular carcinoma, lymphoma, mammary adenocarcinoma, and pulmonary adenoma.^20^, ^21^ While these cases hint at natural carcinogenesis pathways, their molecular drivers—especially *TP53* mutation status—remain largely unexplored, contrasting sharply with established models like mice, dogs, and non‐human primates, where *TP53* mutational landscapes in spontaneous tumors are well documented.^22^, ^23^ Given the role of spontaneous cancers in revealing species‐specific vulnerabilities,^13^ characterizing molecular lesions in tree shrew tumors is essential to evaluate their relevance to human disease.

Here, we address two fundamental gaps to advance the tree shrew as a biomedical model: First (subspecies divergence), we perform the first comparative sequence analysis of *TP53* between *T. b. chinensis* and *T. b. yaoshanensis*, testing for functionally consequential polymorphisms that may impact their comparative utility in cancer research. Second (spontaneous tumor genetics), we report *TP53* sequence variants in two spontaneous tree shrew sarcomas, providing preliminary evidence suggesting p53 pathway involvement in natural carcinogenesis. This integrated approach not only advances the understanding of tree shrew biology but also refines their role as a relevant oncology model and contributes to comparative studies of p53 evolution and function.

## 2 METHODS

### 2.1 Animals and tissue collection

*T. b. chinensis* individuals were sourced from the Kunming Institute of Zoology, Chinese Academy of Sciences. *T. b. yaoshanensis* specimens were captured from the Dayao Mountains in Guangxi, China, and subsequently bred and maintained at the Experimental Animal Center of Guangxi University of Chinese Medicine.

Normal liver tissues were collected from healthy adult individuals of both subspecies after euthanasia. Specifically, normal controls included four *T. b. chinensis* (individual IDs: DX1, DX22, DX16, DXW4) and five *T. b. yaoshanensis* (individual IDs: YS74, YS116, YS7405, YS4, YS175). The sex and age of each individual were recorded (*T. b. chinensis*—DX1: male, 100 months; DX22: female, 54 months; DX16: female, 65 months; DXW4: female, 123 months; *T. b. yaoshanensis*—YS74: female, 91 months; YS116: male, 111 months; YS7405: male, 86 months; YS4: female, 64 months; YS175: female, 105 months). Tissues were rapidly frozen in liquid nitrogen and stored at −80°C for subsequent genomic RNA extraction.

In addition, two spontaneous tumor samples (designated YS186 and YS100) were obtained from *T. b. yaoshanensis* individuals during routine health monitoring. The tumor‐bearing individuals were: YS186 (female, 104 months) and YS100 (male, 127 months). These tumors were excised, divided into portions for histopathological examination and molecular analysis, and processed accordingly: one portion was fixed in 10% neutral buffered formalin for histology, and another was snap‐frozen for RNA extraction.

All animal procedures were approved by the Guangxi University of Chinese Medicine Institutional Animal Ethical and Welfare Committee (Approval No. DW20230519‐638).

### 2.2 Subspecies identification

Subspecies identification was performed using an integrative morphological and molecular approach.

#### 2.2.1 Morphological analysis

Individuals of *T. b. yaoshanensis* and *T. b. chinensis* can be distinguished based on key diagnostic characteristics. *T. b. yaoshanensis* exhibits a larger body size, whereas *T. b. chinensis* has a comparatively smaller body size. The two subspecies can also be differentiated by craniodental measurements and pelage coloration, as established in previous studies by Wang.^17^

#### 2.2.2 Molecular validation

As described in a previously published study,^18^ the mitochondrial genomes of *T. b. yaoshanensis* and *T. b. chinensis* were amplified, compared, and used to construct a phylogenetic tree. Phylogenetic analysis confirmed distinct clustering differences between the two subspecies.

### 2.3 *TP53* gene analysis in subspecies

Total RNA was extracted from frozen normal liver tissues of the nine individuals (YS4, YS74, YS116, YS175, YS7405, DX22, DX16, DXW4, DX1) using the QIAGEN RNA mini‐kit. cDNA was synthesized by reverse transcription for subsequent PCR amplification and sequencing. The entire coding region of *TP53* was amplified in three overlapping fragments using primers specific for the tree shrew *TP53* gene (see Table S1). PCR products were purified and subjected to bidirectional Sanger sequencing on an ABI 3730xl platform. Sequences were aligned to the reference (KF921494.1) to identify subspecies‐specific variants. Amino acid sequences were inferred and aligned with orthologs from other species using Clustal Omega. Structural models were generated via SWISS‐MODEL using human p53 (PDB ID: 7ezj) as a template. Functional impacts of non‐synonymous variants were predicted using SIFT and PROVEAN.

### 2.4 Histopathological diagnosis of spontaneous tumors

Immunohistochemical staining was performed on formalin‐fixed, paraffin‐embedded sections from spontaneous tumor samples (YS186 and YS100, both derived from *T. b. yaoshanensis*) following standard protocols. These included deparaffinization, rehydration, antigen retrieval, and blocking of endogenous peroxidase, then incubation with primary antibodies, followed by HRP‐conjugated secondary antibody incubation, DAB detection, and hematoxylin counterstaining, with negative controls omitting the primary antibody.

The following primary antibodies were used: Vimentin (Abcam, clone VI‐10, 1:250), S‐100 (Abcam, clone EP1576Y, 1:100), Ki‐67 (Abcam, clone SP6, 1:250), CK‐P (MCE, polyclonal, 1:250), SMA (Abcam, clone 1A4, 1:100), Desmin (Abcam, clone Y66, 1:100), CD34 (Abcam, clone EP373Y, 1:2500), GCDFP‐15 (Abcam, clone EP1582Y, 1:100), ER (Abcam, clone EPR4097, 1:250), and GATA3 (Thermo Fisher Scientific, polyclonal, 1:250). Antigen retrieval was performed using citrate buffer (pH 6.0) or EDTA buffer (pH 8.0) as appropriate for each antibody. All secondary antibodies were HRP‐conjugated (Abcam, polyclonal, 1:250), and sections were counterstained with hematoxylin.

### 2.5 Tumor *TP53* variant analysis

To investigate whether *TP53* alterations might be present in spontaneous tree shrew tumors, we analyzed the entire coding region of *TP53* from the two tumor samples (YS186 and YS100). Because both tumor‐bearing animals were returned to their cages for continued postoperative care after tumor resection (in compliance with institutional animal welfare guidelines), no matched normal tissue was available for comparison. Instead, we used the wild‐type *TP53* sequence of *T. b. yaoshanensis* established from healthy individuals (YS175; GenBank PX411279) as a reference.

Total RNA was extracted from frozen tumor tissues (YS186 and YS100) using the QIAGEN RNA mini‐kit (Qiagen, Germany). cDNA was synthesized by reverse transcription. The *TP53* coding region was amplified using primers specific for the tree shrew *TP53* gene (see Table S1). The PCR products were cloned into the pMD‐19 T vector (Takara, China). For each tumor, 8 independent clones were selected and subjected to bidirectional Sanger sequencing on an ABI 3730xl platform. A sequence variant was considered genuine when it was present in at least three independent clones and was absent from the wild‐type reference sequence of the same subspecies. SIFT was used to predict the functional impact of missense variants.

## 3 RESULTS

### 3.1 Integrative taxonomy confirms subspecies identity

Through integrated morphological and molecular analysis, we confirmed the subspecies identity of all *Tupaia belangeri* specimens used in this study. Individuals of *T. b. yaoshanensis* were identified by larger body size (~168.9 g, ~189.7 mm), while *T. b. chinensis* exhibited a smaller body size (~127.8 g, ~167.0 mm), along with distinct craniodental features and pelage coloration, consistent with established diagnostic criteria (Table S2).^17^ Based on our previous study,^18^ molecular validation was performed using the complete mitochondrial genome sequences. Phylogenetic analysis revealed that, while all tree shrew subspecies formed a monophyletic clade closely related to Lagomorpha, *T. b. yaoshanensis* and *T. b. chinensis* were distinctly separated into two terminal branches with high bootstrap support. This clear phylogenetic separation between the two subspecies confirms their genetic differentiation and is consistent with their morphological distinctions.

### 3.2 Comparative analysis of *TP53* gene sequences in tree shrew subspecies

Through comprehensive sequencing of wild‐type *TP53* cDNAs cloned from normal liver tissues of *T. b. chinensis* (individuals: DX1, DX22, DX16, DXW4) and *T. b. yaoshanensis* (individuals: YS7405, YS74, YS4, YS116, YS175), we determined the complete coding nucleotide and deduced amino acid sequences for both subspecies. The *TP53* coding sequences were amplified, cloned into pMD‐19 T vectors, and subjected to bidirectional sequencing.

Comparative analysis revealed a high degree of sequence conservation between the two subspecies, with 99.5% nucleotide identity in the *TP53* coding sequence. A detailed nucleotide alignment illustrating this conservation and the specific variations is provided in Figure S1 1–8. The nucleotide homology of tree shrew *TP53* to human (approximately 85%; AB082923) was higher than that of mouse (80.4%), a finding consistent with the phylogenetic position of Scandentia as more closely related to Primates than to Rodentia. Despite the overall conservation, putative subspecies‐specific nucleotide differences were identified between *T. b. yaoshanensis* and *T. b. chinensis* (Table 1).

**TABLE 1:** Fixed nucleotide and amino acid differences in *TP53* between tree shrew subspecies.

P53 codons (nucleotide position)			Amino acid			Mutation type
*T. b. yaoshanensis*	*T. b. chinensis*	Homo sapiens	*T. b. yaoshanensis*	*T. b. chinensis*	Homo sapiens	
GGU (125)	GAU (125)	GAU (125)	Gly (G)	Asp (D)	Asp (D)	Non‐synonymous
ACG (213)	ACA (213)	ACA (243)	Thr (T)	Thr (T)	Thr (T)	Synonymous
CCA (354)	CCG (354)	CCU (384)	Pro (P)	Pro (P)	Pro (P)	Synonymous
GCA (579)	GCG (579)	GUG (609)	Ala (A)	Ala (A)	Val (V)	Synonymous^a^
CGG (816)	CGA (816)	CGG (846)	Arg (R)	Arg (R)	Arg (R)	Synonymous
CUA (993)	CUG (993)	UUC (1023)	Leu (L)	Leu (L)	Phe (F)	Synonymous^a^

^a^ Synonymous within tree shrew subspecies; non‐synonymous compared to human.

Cross‐species comparison with human *TP53* (Table 1) revealed a conserved pattern. The most notable subspecies divergence was A125G in *T. b. yaoshanensis*, causing an aspartate‐to‐glycine substitution at position 42 (D42G) within TAD1, a region critical for MDM2 binding. SIFT and PROVEAN predicted D42G as tolerated/neutral (SIFT: 0.21; PROVEAN: 0.126).

Beyond this subspecies‐specific variation, we observed broader evolutionary patterns. Three of the six identified variable sites (positions 213, 354, and 816) are synonymous within tree shrews, and the encoded amino acids (Thr71, Pro118, Arg272) are conserved with humans, underscoring strong purifying selection. However, two notable exceptions were identified: at codon 579, both tree shrew subspecies encode alanine (Ala193), whereas humans encode valine (Val203); similarly, at codon 993, tree shrews encode leucine (Leu331), contrasting with phenylalanine (Phe341) in humans. These findings indicate specific residues where the tree shrew lineage has diverged from humans.

Importantly, both tree shrew subspecies maintain strict conservation at key residues equivalent to human cancer hotspot mutations (R175, R249, R273, R282), with no amino acid variations at the corresponding positions (tree shrew sites 165, 239, 263, and 272) (Figure 1). This conservation pattern underscores the functional criticality of these sites across mammalian species.

**FIGURE 1:**
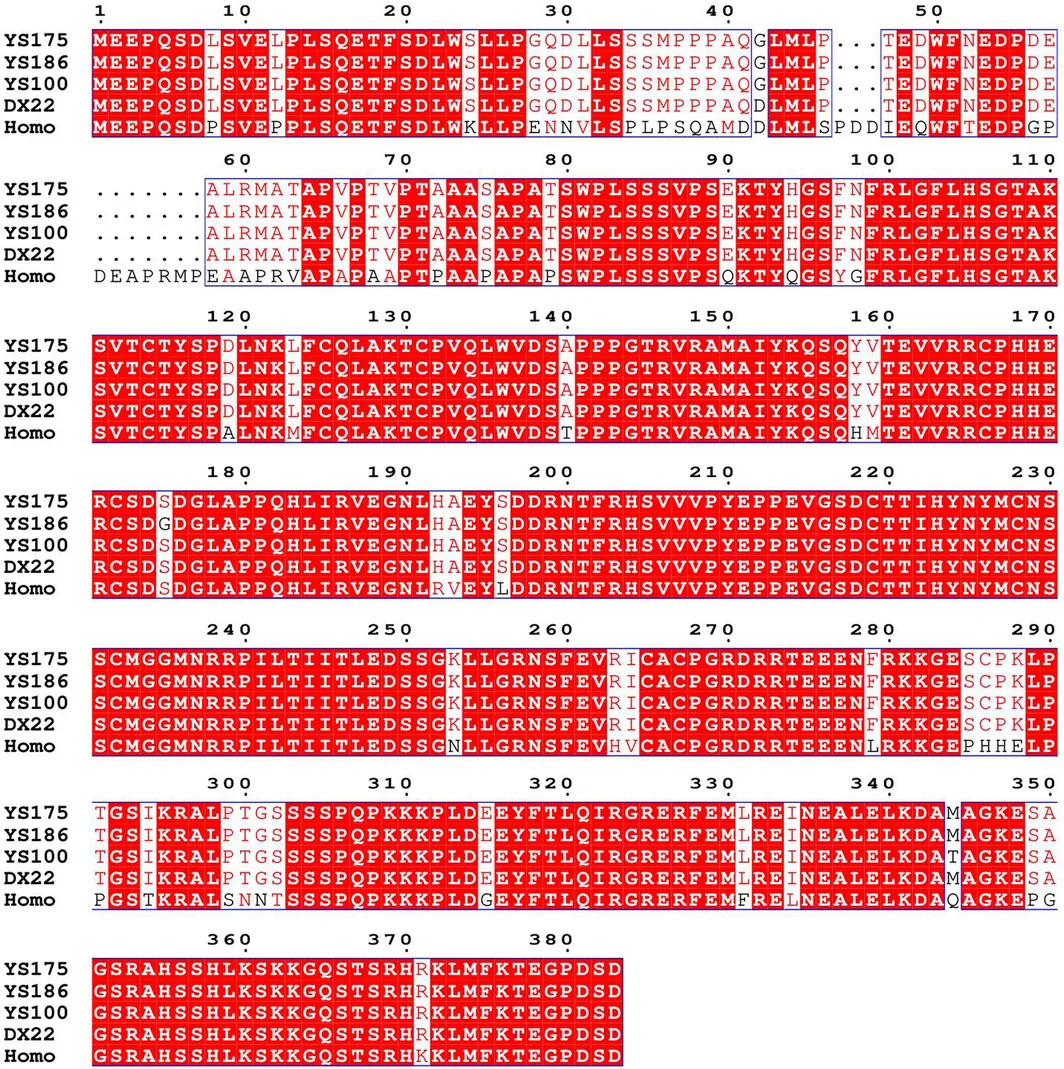
Comparison of the amino acid sequences of tree shrew and human p53 proteins. Identical residues are marked with red boxes; dots indicate gaps (deleted amino acids).

### 3.3 Structural modeling and comparative analysis of tree shrew and human p53 tertiary structure

To assess the structural implications of the sequence variations identified, we generated a tertiary structure model of tree shrew p53 using SWISS‐MODEL with human p53 (Accession: AB082923) as a template (Figure 2). Structural superposition revealed a root‐mean‐square deviation (RMSD) of 0.6 Å between the tree shrew (Figure 2A) and human p53 structures (PDB ID: 7ezj) (Figure 2B), indicating a high degree of structural conservation (Figure 2C).

**FIGURE 2:**
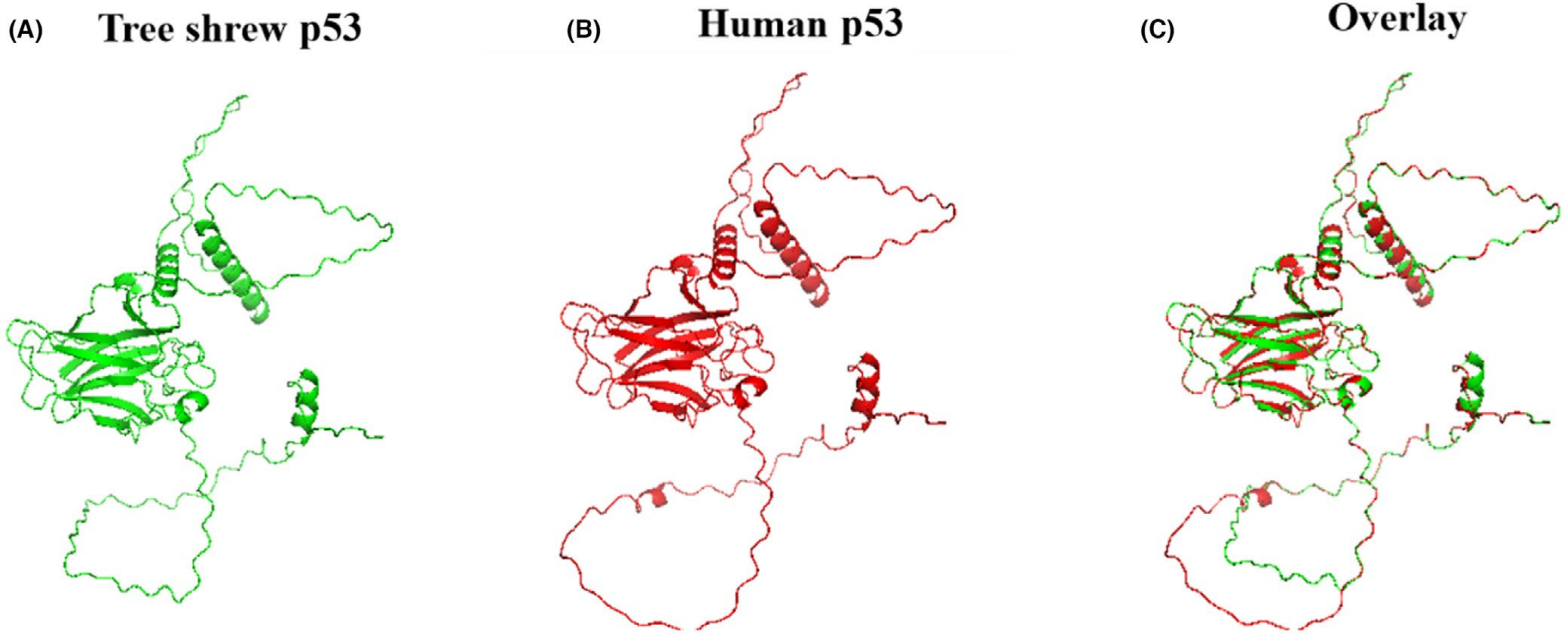
Tertiary structural models of tree shrew and human p53 proteins. (A) Tree shrew (green) p53 proteins. (B) Human (red) p53 proteins. (C) Overall structure superposition of tree shrew (green) and human (red) p53.

The core DNA‐binding domain exhibited particularly strong conservation, with key functional motifs maintaining nearly identical conformations between species: the DNA‐binding interface formed by the L2/L3 loops and the loop‐sheet‐helix motif were structurally aligned; zinc‐coordinating residues (Cys176, His179, Cys238, Cys242 in human; corresponding to tree shrew positions 166, 169, 228, 232) maintained identical spatial configuration; and residues critical for DNA contact (e.g., human Arg248→tree shrew Arg238) were conserved.

As anticipated from sequence analysis, minor structural variations were observed in the N‐terminal transactivation domain (TAD1) and the C‐terminal regulatory domain, regions known to exhibit higher sequence divergence across species. Structural modeling of the D42G substitution in *T. b. yaoshanensis* confirmed that this variation occurs within a flexible loop region of TAD1, replacing a charged aspartic acid with a neutral glycine without perturbing the overall α‐helix H1 or domain structure. This structural observation aligns with the computational predictions of neutral functional impact for this substitution.

### 3.4 Pathological characterization and *TP53* variant analysis of spontaneous tumors in *T. b. yaoshanensis*

#### 3.4.1 Histopathological and immunophenotypic characterization

##### Macroscopic findings

YS186 was a firm, poorly circumscribed subcutaneous mass (~3.0 × 2.5 × 2.0 cm) on the left hindlimb root with pale yellow cut surface. YS100 was a gelatinous subcutaneous mass (~2.0 × 1.0 × 1.5 cm) on the right forelimb infiltrating adjacent fascia. No metastases were observed.

##### Histopathological and immunohistochemical findings

YS186 was diagnosed as pleomorphic liposarcoma, showing infiltrative growth and two components: pleomorphic sarcomatous foci and lipoblastic regions with multivacuolated cytoplasm (Figure 3A,B). Immunohistochemistry showed diffuse Vimentin, focal S‐100, Ki‐67 ~ 20%, and negativity for epithelial markers (CK‐P, GCDFP‐15, ER, GATA3) (Figure 3C–I). YS100 was an infiltrative malignant mesenchymal tumor with epithelioid cells, multinucleated giant cells, myxoid stroma, and focal lipoblast‐like cells (Figure 4A,B). Immunohistochemistry showed Vimentin+ and Ki‐67 ~ 20%, but was negative for S‐100, CK‐P, SMA, Desmin, and CD34 (Figure 4C–I). However, as most antibodies were raised against human antigens, their lack of reactivity may reflect limited cross‐species recognition. Thus, while histology suggested a myxoid sarcoma (myxofibrosarcoma or dedifferentiated liposarcoma), definitive subclassification could not be established. Both tumors showed infiltrative growth, high nuclear grade, and elevated mitotic activity.

**FIGURE 3:**
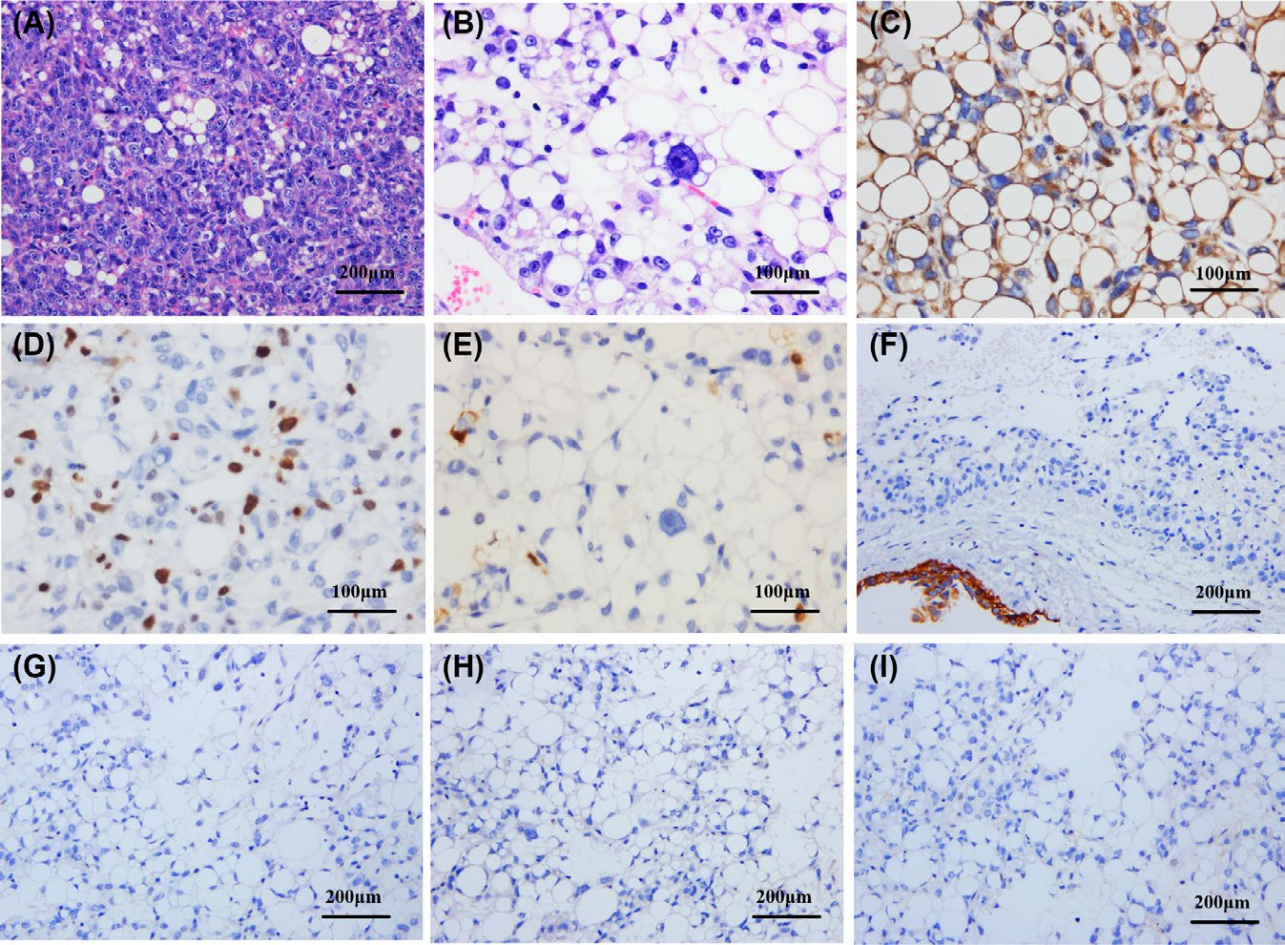
Histopathological and immunohistochemical features of the spontaneous tumor (YS186) in *T. b. yaoshanensis*. (A) Pleomorphic sarcoma area composed of epithelioid cells with significant nuclear atypia and prominent nucleoli (×200). (B) Lipoblasts showing marked variation in cell size and multivacuolated cytoplasmic lipid droplets (×400). (C) Vimentin immunohistochemistry demonstrating diffuse membranous and cytoplasmic positivity in lipoblasts (×400). (D) Ki‐67 immunohistochemistry showing focal nuclear positivity in lipoblasts (×400). (E) S‐100 immunohistochemistry showing focal nuclear positivity in lipoblasts (×400). (F) Tumor cells are negative for CK‐P immunohistochemistry (×200). (The adjacent ductal epithelium in the lower left corner serves as an internal positive control.) (G) Tumor cells are negative for GCDFP‐15 immunohistochemistry (×200). (H) Tumor cells are negative for ER immunohistochemistry (×200). (I) Tumor cells are negative for GATA3 immunohistochemistry (×200).

**FIGURE 4:**
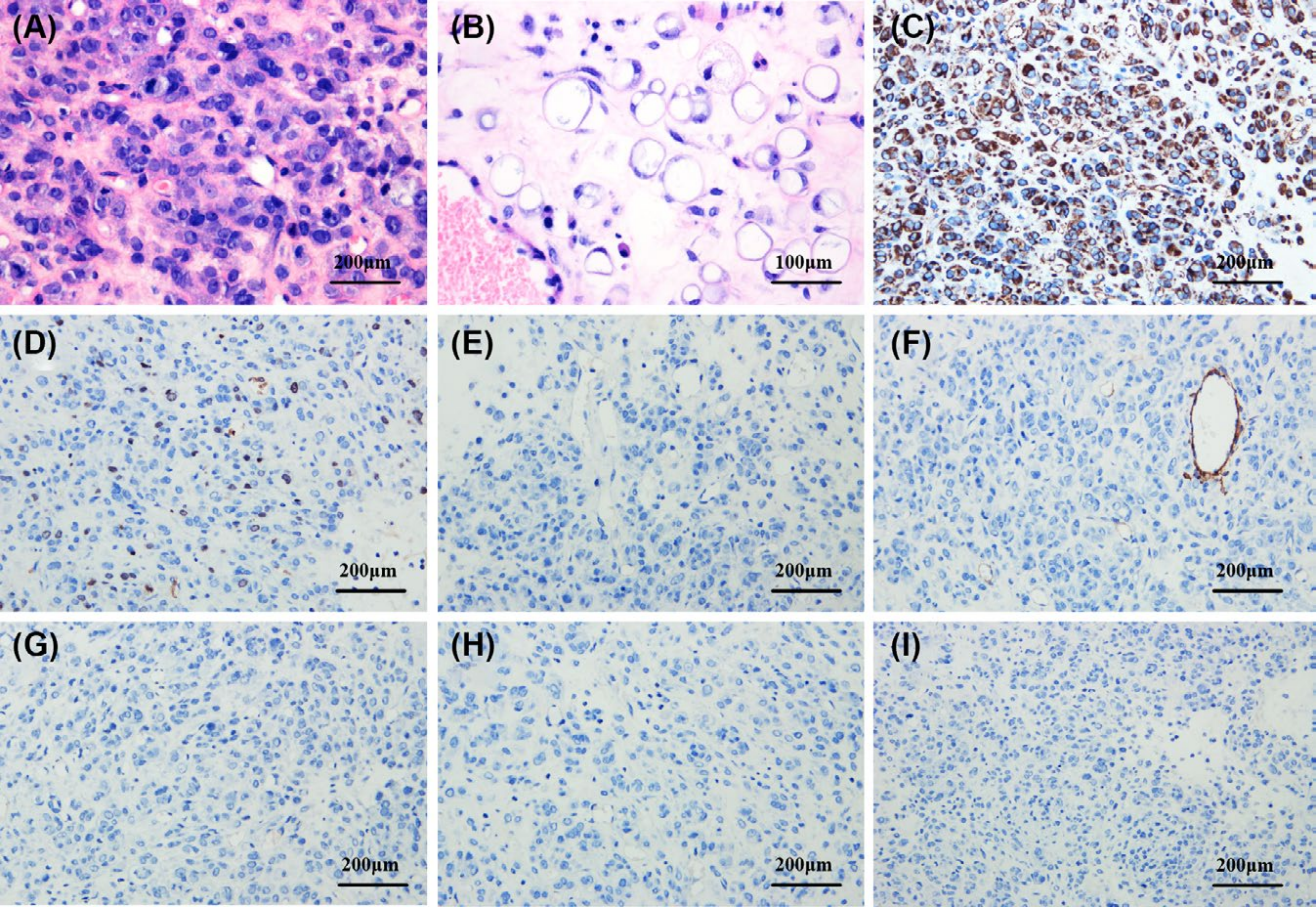
Histopathological and immunohistochemical features of the spontaneous tumor (YS100) in *T. b. yaoshanensis*. (A) Epithelioid tumor cells exhibiting nuclear atypia and readily identifiable mitotic figures (×200). (B) Cells of varying size resembling adipocytes (×200). (C) Vimentin immunohistochemistry showing diffuse cytoplasmic positivity in tumor cells (×200). (D) Ki‐67 immunohistochemistry showing focal nuclear positivity in tumor cells (×200). (E) Tumor cells are negative for S‐100 immunohistochemistry (×200). (F) Tumor cells are negative for SMA immunohistochemistry (×200). (The smooth muscle in the vessel wall serves as an internal positive control). (G) Tumor cells are negative for CK‐P immunohistochemistry (×200). (H) Tumor cells are negative for Desmin immunohistochemistry (×200). (I) Tumor cells are negative for CD34 immunohistochemistry (×200).

#### 3.4.2 Identification of *TP53* variants in spontaneous tumors

Using clonal sequencing and comparing to the wild‐type reference (YS175; GenBank PX411279), we identified the following variants summarized in Table 2. In YS186 (pleomorphic liposarcoma), three variants were identified: a missense A→G transition at nucleotide position 523 (codon 175), resulting in a serine‐to‐glycine substitution (Ser175Gly) within the DNA‐binding domain (DBD); a silent C→T transition at nucleotide 711 (codon 237, Asn237Asn); and a silent T→C transition at nucleotide 975 (codon 325, Arg325Arg). In YS100 (myxofibrosarcoma), two variants were identified: a silent G→A transition at nucleotide 231 (codon 77, Pro77Pro) within the transactivation domain 1 (TAD1), and a missense T→C transition at nucleotide 1031 (codon 344), resulting in a methionine‐to‐threonine substitution (Met344Thr) in the tetramerization domain.

**TABLE 2:** *TP53* gene variants identified in spontaneous tumors of tree shrews.

Sample	Tumor type	Nucleotide position	Codon	Nucleotide change	Amino acid change	Variant type	Functional domain
YS186	Pleomorphic liposarcoma	523	175	A→G	Ser175Gly	Missense	DNA‐binding
		711	237	C→T	Asn237Asn	Silent	DNA‐binding
		975	325	T→C	Arg325Arg	Silent	Not in core domain
YS100	Myxofibrosarcoma	231	77	G→A	Pro77Pro	Silent	Transactivation domain 1 (TAD1)
		1031	344	T→C	Met344Thr	Missense	Tetramerization

##### Computational prediction

SIFT predicted both missense variants as tolerated (Ser175Gly: 0.61; Met344Thr: 0.77; damaging threshold ≤0.05), suggesting they are unlikely to severely impair p53 function based on sequence conservation alone. Functional studies are needed to assess their biological significance.

## 4 DISCUSSION

The present study provides an integrated analysis of p53 in the tree shrew, spanning comparative genomics across subspecies and sequence variant profiling in spontaneous tumors. Our findings delineate a complex landscape of evolutionary conservation, natural variation, and tumor‐associated *TP53* sequence changes in *Tupaia belangeri*, with implications for refining this species as a biomedical model.

Our comparative analysis between *T. b. yaoshanensis* and *T. b. chinensis* reveals the evolutionary forces shaping the *TP53* gene. The abundance of synonymous substitutions, particularly A↔G transitions, alongside the fixed non‐synonymous D42G variant in TAD1, primarily reflects the actions of genetic drift and neutral evolution in geographically isolated populations. The D42G substitution was computationally predicted to be neutral, consistent with its location in a flexible loop region of TAD1 where structural plasticity is tolerated. This contrasts with the absolute conservation we observed at residues equivalent to human cancer hotspots (e.g., R175, R249), illustrating the intense purifying selection that maintains core functions, such as DNA‐binding integrity, across mammalian evolution.

Beyond subspecies divergence, our cross‐species comparison identified fixed amino acid differences between tree shrew and human p53 (e.g., Ala193Val, Leu331Phe). While these residues lie outside the core DNA‐binding domain, their evolutionary divergence cautions against directly extrapolating all aspects of p53 regulation. Nevertheless, the high degree of structural conservation, particularly within the DBD (RMSD = 0.6 Å), and the preservation of key functional motifs suggest that the fundamental tumor‐suppressive machinery is likely conserved.

### 4.1 Implications of the identified *TP53* variants

Both missense variants were predicted tolerated by SIFT (Ser175Gly: 0.61; Met344Thr: 0.77), suggesting they are unlikely to severely impair p53 function based on sequence conservation alone. Several interpretations are possible: they may be incidental passenger mutations; they could have subtle effects not captured by computational tools (e.g., altered stability or co‐factor interactions); or other genetic/epigenetic alterations may drive tumorigenesis while these variants are bystanders. We cannot conclude these variants are causative, but their presence in two spontaneous sarcomas—including Met344Thr in the tetramerization domain—warrants further functional investigation.

### 4.2 Implications for the tree shrew as a cancer model

Our findings provide multi‐layered information supporting the tree shrew, particularly *T. b. yaoshanensis*, as a potential animal model for cancer research. The high degree of *TP53* sequence and structural conservation with humans, especially at critical cancer hotspot residues, suggests that functional insights gained from tree shrew studies may be relevant to human biology. The detection of *TP53* variants in spontaneous sarcomas indicates that this tumor suppressor pathway may be involved in natural carcinogenesis in this species. However, given that the identified missense variants were predicted to be tolerated, their functional significance remains uncertain, and further studies are needed to clarify the role of *TP53* in tree shrew tumorigenesis.

### 4.3 Limitations

First, sample size is limited to two tumors. Second, no matched normal tissue was available (animals not euthanized after tumor resection), precluding definitive confirmation of somatic origin. Third, SIFT predictions suggest both missense variants are tolerated, but functional assays are needed. Fourth, p53 IHC was not possible due to lack of cross‐reactive antibodies. Our team is currently investigating *CREPT* conservation in tree shrews, with preliminary results indicating high conservation; other cancer‐related genes will be examined in future studies. Fifth, YS100 immunophenotyping was limited by human‐derived antibodies; lack of reactivity for lineage markers may reflect cross‐species issues rather than true antigen absence, precluding definitive subclassification. Sixth, silent variants may affect splicing, but this was not investigated. Future studies with larger cohorts, paired normal tissues, tree shrew‐specific antibodies, and functional validation are needed.

## 5 CONCLUSION

In conclusion, our integrated study delineates the genetic landscape of *TP53* in tree shrews, revealing evolutionary constraints, natural polymorphisms, and tumor‐associated sequence variants. While the functional significance of the identified missense variants remains to be determined, this work provides a foundation for future investigations into p53 biology and cancer modeling using the tree shrew.

## AUTHOR CONTRIBUTIONS

**Yingying Cao:** Formal analysis; methodology; data curation. **Haibo Tang:** Supervision. **Liang Liang:** Formal analysis. **Weijian Huang:** Methodology; project administration. **Jing Leng:** Conceptualization; funding acquisition.

## FUNDING INFORMATION

The Foundation of Guangxi Educational Committee, Grant/Award Number: 2023KY0321; The School‐level scientific research project of Guangxi University of Chinese Medicine, Grant/Award Number: 2023QN002.

## CONFLICT OF INTEREST STATEMENT

The authors declare no competing or financial interests.

## ETHICS STATEMENT

All animal procedures were approved by the Guangxi University of Chinese Medicine Institutional Animal Ethical and Welfare Committee (approval no.: DW20230519‐638). All experiments were performed in accordance with the institution's guidelines and the National Standards for the Welfare of Laboratory Animals (GB/T 35892‐2018) of the People's Republic of China.

## Supporting information


**Supplementary Figure S1.** 1–8 Nucleotide sequence alignment of the *TP53* coding region among tree shrew individuals and human reference. Sequences were obtained from *T. b. yaoshanensis* (YS175, YS74, YS4, YS116, YS7405, YS186, YS100) and *T. b. chinensis* (DX22, DX16, DXW4, DX1), with *Homo sapiens* (AB082923.1) included for comparison. The alignment spans the complete coding region (nucleotide positions 1 to 1182). Gaps introduced to optimize alignment are represented by short dashes (‐). This alignment illustrates the high nucleotide sequence conservation (99.5% identity) between the two subspecies and the specific nucleotide differences listed in Table 1.

**Table S1.** Primer design of the ts*TP53*.

**Table S2.** Comparison of phenotypic characteristics between the *T. b. yaoshanensis* and the *T. b. chinensis*.

## Data Availability

The sequence and annotation of the *TP53* genome of *T. b. yaoshanensis* 175 were deposited into GenBank and assigned an accession number PX411279.
