# Supplementary material for: Comparative analysis of TP53 gene in Tupaia belangeri subspecies (Tupaia belangeri yaoshanensis vs. Tupaia belangeri chinensis) and identification of mutations in spontaneous tumor cases
Source: Animal Model Exp Med. 2026 Aug 17;9(9):1901–9. doi: 10.1002/ame2.70260 (PMC13479160; doi:10.1002/ame2.70260)
Supplement: Supplementary file 1 [file AME2-9-1901-s001.docx]

Table S1 Primer design of the ts*TP53*

| Primers | Primer sequence | Location |
| --- | --- | --- |
| ts*tp53*-1F | GCCATGGAGGAGCCGCAGTC | 1-355 |
| ts*tp53*-1R | GGAATACGTGCAGGTGACAGA |  |
| ts*tp53*-2F | GGACCTTCTGTCCTCCTCGAT | 87-1150 |
| ts*tp53*-2R | AGTCTGAGTCAGGTCCCTCT |  |
| ts*tp53*-3F | CGACTGGTTCCAGCTCTTCTC | 900-1244 |
| ts*tp53*-3R | GCGGGGGTTTAAGGACCAGAA |  |
