# Supplementary material for: Comparative analysis of TP53 gene in Tupaia belangeri subspecies (Tupaia belangeri yaoshanensis vs. Tupaia belangeri chinensis) and identification of mutations in spontaneous tumor cases
Source: Animal Model Exp Med. 2026 Aug 17;9(9):1901–9. doi: 10.1002/ame2.70260 (PMC13479160; doi:10.1002/ame2.70260)
Supplement: Supplementary file 2 [file AME2-9-1901-s002.docx]

Table S2. Comparison of phenotypic characteristics between the *T. b. yaoshanensis* and the *T. b. chinensis*

| Subspecies | Number | Gender | Age(year) | Body weight (g) | Body length (mm) | Tail length (mm) | Ear length (mm) | Skull length (mm) |
| --- | --- | --- | --- | --- | --- | --- | --- | --- |
| *T. b. yaoshanensis* | YS74 | Female | 7 | 178 | 205 | 175 | 15 | 50 |
|  | YS4 | Female | 5 | 168 | 179 | 174 | 13 | 49 |
|  | YS116 | male | 9 | 180 | 204 | 176 | 15 | 51 |
|  | YS175 | Female | 8 | 194 | 206 | 176 | 16 | 51 |
|  | YS7405 | male | 7 | 171 | 181 | 173 | 13 | 50 |
|  | YS186 | Female | 8 | 151 | 178 | 174 | 13 | 49 |
|  | YS100 | male | 10 | 140 | 175 | 172 | 12 | 49 |
|  | Mean ± SD (*n* = 7) | | | 168.9 ± 18.9 | 189.7 ± 13.7 | 174.3 ± 1.5 | 13.9 ± 1.5 | 49.9 ± 0.9 |
| *T. b. chinensis* | DX22 | Female | 4 | 128 | 165 | 169 | 15 | 46 |
|  | DX16 | Female | 5 | 135 | 166 | 169 | 15 | 47 |
|  | DXW4 | Female | 10 | 118 | 167 | 168 | 15 | 45 |
|  | DX1 | male | 8 | 130 | 170 | 170 | 16 | 47 |
|  | Mean ± SD (*n* = 4) | | | 127.8 ± 7.1 | 167.0 ± 2.2 | 169.0 ± 0.8 | 15.3 ± 0.5 | 46.3 ± 0.9 |
